# RAW Image Reconstruction Using a Self-contained sRGB–JPEG Image with Small Memory Overhead

**DOI:** 10.1007/s11263-017-1056-0

**Published:** 2017-12-18

**Authors:** Rang M. H. Nguyen, Michael S. Brown

**Affiliations:** 1 School of Computing, National University of Singapore, Singapore, Singapore; 2 Lassonde School of Engineering, York University, Toronto, Canada

**Keywords:** Radiometric calibration, In-camera image processing, Raw image reconstruction

## Abstract

Most camera images are saved as 8-bit standard RGB (sRGB) compressed JPEGs. Even when JPEG compression is set to its highest quality, the encoded sRGB image has been significantly processed in terms of color and tone manipulation. This makes sRGB–JPEG images undesirable for many computer vision tasks that assume a direct relationship between pixel values and incoming light. For such applications, the RAW image format is preferred, as RAW represents a minimally processed, sensor-specific RGB image that is linear with respect to scene radiance. The drawback with RAW images, however, is that they require large amounts of storage and are not well-supported by many imaging applications. To address this issue, we present a method to encode the necessary data within an sRGB–JPEG image to reconstruct a high-quality RAW image. Our approach requires no calibration of the camera’s colorimetric properties and can reconstruct the original RAW to within 0.5% error with a small memory overhead for the additional data (e.g., 128 KB). More importantly, our output is a fully self-contained 100% compliant sRGB–JPEG file that can be used as-is, not affecting any existing image workflow—the RAW image data can be extracted when needed, or ignored otherwise. We detail our approach and show its effectiveness against competing strategies.

## Introduction

The vast majority of images used in computer vision and image processing applications are 8-bit standard RGB (sRGB) images, typically saved using the JPEG compression standard. Virtually all imaging application workflows support sRGB and JPEG images. There are many drawbacks, however, when working with sRGB images. For example, it is well known that sRGB images are processed by a number of nonlinear operations that make it difficult to relate the sRGB values back to scene radiance (Chakrabarti et al. 2014; Debevec and Malik 2008; Grossberg and Nayar 2003; Kim et al. 2012; Mann et al. 1995; Mitsunaga and Nayar 1999).

Most cameras now allow images to be saved in a RAW image format that is an uncompressed, minimally processed image format representing the response from the camera sensor. RAW has many advantages over sRGB, including linear response to scene radiance, wider color gamut, and higher dynamic range (generally 12–14 bits). Not surprisingly, RAW is desirable for many computer vision applications, such as photometric stereo, image restoration (e.g., deblurring), white balance, and more. RAW is also preferred by photographers as it allows flexibility for post-processing manipulation. One of the major drawbacks to using RAW images is that RAW files take up significantly more space than their sRGB counterpart. In addition, the vast majority of existing image-based applications are designed to work with 8-bit sRGB images. Images saved in RAW format must undergo some intermediate process to convert them into sRGB to be compatible with many existing applications.

Given the utility of RAW image data, there have been a number of approaches to map sRGB images back to their RAW values. Work by Yuan and Sun (2011) demonstrated an effective hybrid-image method that stored a lower-resolution version of the original RAW image (e.g., $\frac{1}{2}$ or $\frac{1}{4}$ resolution) and applied smart upsampling that leveraged the sRGB image. One impetus for Yuan and Sun (2011)’s work is that many cameras now support a small-RAW format that saves the RAW image in either half- or quarter-size resolutions. However, it is important to note that these smaller RAW images still require approximately 1.5–6 MB to store. Other closely related work (Xiong et al. 2012; Chakrabarti et al. 2014; Kim et al. 2012; Lin et al. 2011, 2012) used a calibration procedure to parameterize a model of the onboard camera processing pipeline in order to reverse sRGB values back to their RAW values. While this calibrated data is generally smaller than the 1.5–6 MB needed using the small-RAW strategy, these methods still have a drawback in that the additional data needs to be saved separately. In addition, the calibration procedure used by these methods needs to be performed for several different camera settings. Moreover, the model parameters are estimated in order to minimize reconstruction errors of a wide range of colors, because it is assumed that the original RAW is not given when converting back from sRGB. In our problem scenario, we have the original RAW image and corresponding sRGB image as input and can therefore estimate model parameters that better fit the given image pair.

**Fig. 1:**
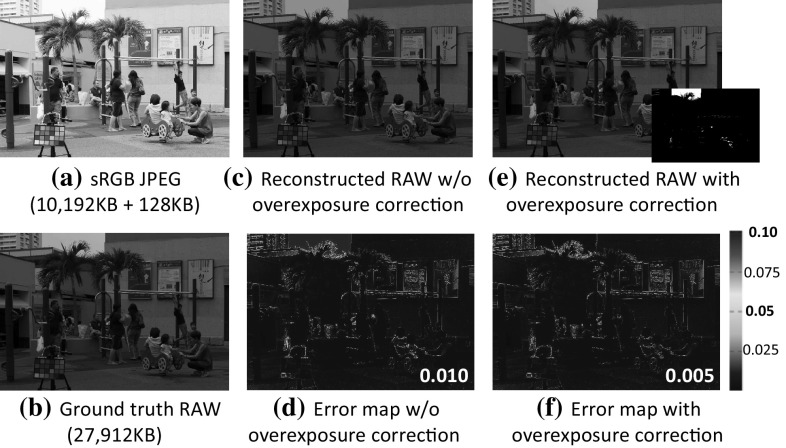
**a** A $5184 \times 3456$ resolution high-quality sRGB–JPEG with the proposed data embedded (original JPEG size of 10,192 KB, new size of 10,320 KB, which contains 64 KB for in-camera pipeline parameters and 64 KB for saturation table). **b** Original RAW image is 27,912 KB. **c** Our reconstructed RAW image using the data in the self-contained sRGB–JPEG without saturation correction. **d** Error map between (**b**) and (**c**). **e** Our reconstructed RAW image with saturation correction (the bottom right is the saturation map where white color indicates saturated pixels). **f** Error map between (**b**) and (**e**). The RMSEs for each method are shown in the bottom right of each error map

The goal of this paper is to compute the necessary data to allow an sRGB–JPEG input to reconstruct its corresponding RAW image. In addition, we want to do this with a small memory overhead that can be embedded within the JPEG image such that it is 100% compatible with the existing JPEG standard. Specifically, we restrict our data to only 128 KB:64 KB for the camera-pipeline parameters and 64 KB for saturation correction. We show that with only 128 KB of data we can reconstruct the original RAW image with an overall error of less than 0.5%. Furthermore, this data can be embedded in two 64 KB JPEG comment fields allowed by the JPEG file format. Figure 1 shows an example of our proposed method’s ability.

*Contribution*  We provide a straightforward and effective procedure to extract the necessary data for reconstructing a RAW image given the corresponding sRGB–JPEG image. As part of this procedure, we describe a fast breadth-first-search octree algorithm for finding the necessary control points to provide a mapping between the sRGB and RAW sensor color spaces. This allows us to effectively encode the nonlinear color space transform between the sRGB and RAW values. Due to the differences in dynamic range between RAW and sRGB, there may be a number of regions in the sRGB image that have been clipped (i.e., one color channel is fully saturated). To this end, we also introduce a strategy to handle these saturated regions with a small memory footprint to allow a better reconstruction. Finally, we describe how to encode the required model data efficiently within text-comment fields supported by the JPEG compression standard. Embedding our data as text-comment fields allows our method to be fully compatible with existing JPEG libraries and workflows. We compare our approach with existing methods and demonstrate the usefulness of the reconstructed RAW on two applications: white-balance correction and image-deblurring.

We note that a shorter conference version of this paper appeared in Nguyen and Brown (2016). Our initial conference paper did not address the problem of saturation. This manuscript addresses this issue and provides additional analysis on the trade-off between using more data (i.e., a larger memory footprint) and the overall image reconstruction quality.

## Related Work

Work related to RAW image reconstruction can be categorized into two areas: (1) radiometric and camera color calibration and (2) methods for image-upsampling.

*Radiometric and color calibration* are methods that aim to compute the necessary mappings to invert the nonlinear transformations applied on board cameras in order to have pixel values that are linear with respect to scene radiance. Conventional radiometric calibration algorithms used multiple images taken with controlled exposures in order to compute the response function of a camera’s output intensity values with respect to the amount of light falling on the sensor. These methods targeted greyscale images (Lin and Zhang 2005) or computed an individual response function per color channel (Debevec and Malik 2008; Grossberg and Nayar 2003; Mann et al. 1995; Mitsunaga and Nayar 1999). The main difference among these methods is the model used to represent the response function—for example, exponentiation (Mann et al. 1995), polynomial (Mitsunaga and Nayar 1999), non-parametric (Debevec and Malik 2008), and PCA-based (Grossberg and Nayar 2003).

These early methods discarded RGB values that were too saturated, treating them as outliers to the radiometric model. Work in Chakrabarti et al. (2009) and Kim et al. (2012) found that these color outliers were due to limitations of existing radiometric models that treated each channel independently. To overcome this, Chakrabarti et al. (2009) proposed a method that used combinations of cross-channel linear transforms with per-channel multivariate polynomials to model the camera color-mapping process. Kim et al. (2012) proposed an in-camera imaging model that introduced an additional 3D gamut mapping step for handling the out-of-gamut colors. Later, Chakrabarti et al. (2014) extended this idea and suggested using uncertainty modeling for handling the quantization of the sRGB colors.

These recent radiometric calibration methods significantly improve the ability to reverse sRGB images back to their RAW values; however, they do have several limitations with respect to the problem addressed in this paper. The first limitation is the need to calibrate the color models for a given camera. As discussed by Kim et al. (2012), this involves computing multiple parameterized models for different camera settings. For example, each picture style (e.g., landscape, portrait, vivid, standard, neutral) requires its own set of parameters. As a result, a single camera would have numerous color mappings that would need to be saved. Such camera calibration is burdensome. Another drawback is the parameterized models need to be saved offline as an additional file and are still too large to be suitable to be embedded into a JPEG image. Finally, this type of color calibration is done in a generic manner, attempting to minimize reconstruction errors over a wide range of input images. This is because these methods assume that the original RAW image is unknown, and only an sRGB image is available as input. In our application, we have access to the original RAW and sRGB image pair and therefore can compute model parameters specific to the input pair.

*Image upsampling* is a method that attempts to increase the resolution, or quality, of an image. Representative work includes interpolation-based methods (Hou and Andrews 1978; Thévenaz et al. 2000), edge-based methods (Dai et al. 2007; Fattal 2007; Sun et al. 2008), and example-based methods (Freeman et al. 2000; Glasner et al. 2009). These methods leverage a dictionary of image patches learned from high-quality images that are used to guide the upsampling process. Work by Yuan and Sun (2011) presented work that is similar to the problem addressed in this paper. In particular, Yuan and Sun (2011) proposed a hybrid-image method that stored a low-resolution version of the RAW image together with the original high-resolution sRGB–JPEG image. The low-resolution RAW image was upsampled to have the same resolution as the sRGB image by using the sRGB image to guide the upsampling process. The RAW images used in this work were one half or one quarter the size of the original RAW image. While these small RAW images are smaller than the original RAW image, they still require 1.5–6 MB to store. Also, like the work of Kim et al. (2012) and Chakrabarti et al. (2014), this approach requires additional data to be stored separately from the JPEG image in order to perform upsampling.

The work in this paper is distinguished from prior work in two ways. First, the aim here is to embed the necessary information for RAW reconstruction inside the existing sRGB–JPEG image. This is more practical than requiring the user to maintain a companion file containing the necessary data for reconstruction (e.g., small RAW file or camera calibration data). Second, unlike the radiometric calibration approaches, our approach does not require a calibration procedure involving multiple images. Instead, we need only to estimate a mapping between the given sRGB and RAW image pair provided by the camera. To this end, our goal is to efficiently estimate this necessary data such that it can fit inside text-based comments that can be embedded in the JPEG file.

**Fig. 2:**
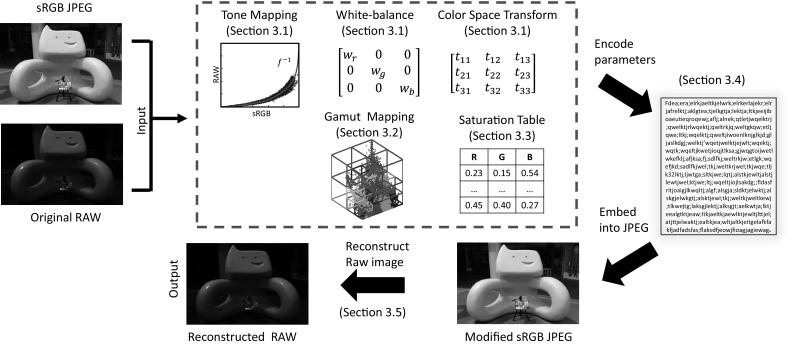
This figure shows an overview of our approach and the corresponding sections in the paper that detail each step

## Proposed Approach

The work by Kim et al. (2012) and Chakrabarti et al. (2014) has shown that the nonlinear processes that convert RAW to sRGB can be described by a number of parameters that model various steps in the onboard camera color-processing pipeline. In particular, there is a white-balance matrix $T_w$ and a color-correction matrix $T_s$ that are first applied. The color-correction matrix is used to convert the camera-specific RAW color space into a device-independent color space. This is followed by a tone map operator *f* applied to all color channels and a gamut-mapping *g* that maps a 3D color value to a new 3D color value. The work in Kim et al. (2012) and Chakrabarti et al. (2014) parameterized these models by using many sRGB–JPEG pairs of color charts under different illuminations captured by the camera with specific settings. This type of parameterization is necessary when the input is an arbitrary sRGB image. We follow a similar procedure, but our problem is simplified as we need only to compute these parameters for a single pair of RAW image *E* and sRGB–JPEG image *I*. In addition, our approach needs to keep the results to within a small memory overhead and embed this as a text comment in the JPEG image.

**Table 1:** This table shows the amount of data allocated to model the camera-pipeline parameters and saturation table (if necessary) for a RAW-sRGB image pair

	Variable	Original size	Type	Storage size (byte)
In-camera pipeline parameters (64 KB)	$T_w^{-1}$	$3 \times 1$	double	24
	$T_s^{-1}$	$3 \times 3$	double	72
	$f^{-1}$	$256 \times 1$	int16	512
	$g^{-1}$	$4728 \times 6$	int16	56,736
Saturation table (64 KB)	*S*	$9557 \times 3$	int16	57,342

*Note* The goal is to keep each part fitted inside 64 KB COM segments of data of JPEG files

Figure 2 illustrates the overall procedure of our method. Our input is the RAW image captured from the camera and the corresponding sRGB–JPEG. We assume the RAW image has been demosaicked. We use the DCRAW utility (Coffin 1997) to perform this task; however, other methods could also be used—for example, Gharbi et al. (2016). The data storage budget for the camera model and saturation table is pre-allocated as shown in Table 1. The total budget for both the camera model parameters and saturation table is less than 128 KB because this data will later be converted to a text format that avoids a 0x00 bit sequence (described in Sect. 3.4). The following sections describe how this data is computed and embedded in the sRGB–JPEG file.

### In-Camera Imaging Model Estimation

**Fig. 3:**
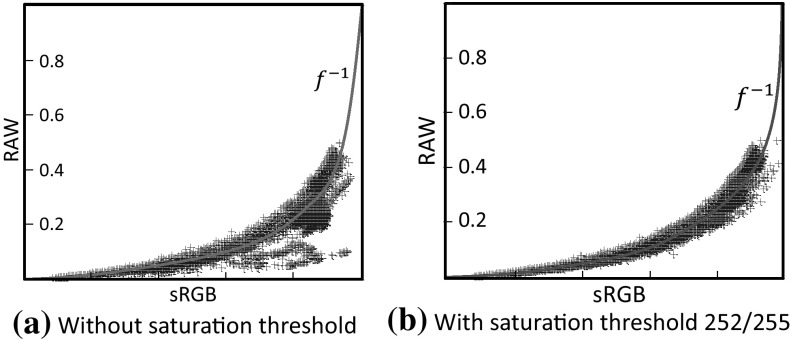
This figure shows an example with and without using a saturation threshold for estimating an inverse tone-curve $f^{-1}$

Our first step is to compute an inverse tone-curve, $f^{-1}$, from an sRGB–JPEG and RAW image pair. This is used to make the sRGB values more linear with respect to the RAW value. We assume that the gamut-mapping procedure affects mainly chromatic colors and therefore first identify pixels containing achromatic colors (i.e., pixels whose RGB values are too close to greyscale) for estimating the tone-curve. Based on this assumption, the sRGB image is converted into the HSV color space, and the color values having saturation channel S less than the threshold of 0.2 are selected. Then the V channel of these colors is used to estimate the inverse tone-curve $f^{-1}$. This curve can be estimated using the spline-fitting technique (Reinsch 1967) as follows:

1 $$\begin{aligned}&f^{-1} = \underset{f^{-1}}{\mathrm {argmin}} \frac{1}{N} \sum \nolimits _{i=1}^{N}{||f^{-1}(I_i) - E_i||^2} + \lambda ||\triangledown ^2 f^{-1}||^2, \nonumber \\&\qquad \qquad \qquad \qquad \quad \quad \qquad \text {s.t}~~ \triangledown ^1 f^{-1} \ge 0 \end{aligned}$$

where *i* is the index to color pixels, *N* is the number of color points after applying a saturation threshold, $\triangledown ^1$ denotes the first derivative, and $\triangledown ^2$ denotes the second derivative. The first term measures the agreement of $f^{-1}$ with the observations, while the second term constrains the smoothness of the curve. The weight $\lambda$ controls the relative contribution of the two ($\lambda =0.1$ for all our examples). There is also a hard constraint that the function is monotonically increasing. It is also worth noting that color values with any channel set close to 255 (e.g., larger than the threshold $\tau = 252/255$) are not used as they represent fully saturated pixels. Figure 3 shows an example with/without using the achromatic threshold for estimating an inverse tone-curve $f^{-1}$.

After the tone-mapping *f* is estimated, sRGB values are converted to linearized sRGB values using $f^{-1}$. As with the tone-curve, the linear color-correction matrix $T_c$ is computed using the color values with low color saturation that are not affected by the gamut mapping. Here, the color correction matrix $T_c$ is the combination of the white-balance matrix $T_w$ and the color space transformation matrix $T_s$. We estimate the matrix $T_c$ that minimizes the following error function:

2 $$\begin{aligned} T_c = \underset{T_c}{\mathrm {argmin}} \sum _{i=1}^{N}{||f^{-1}}(I_i) - T_c E_i||^2. \end{aligned}$$

Note that most consumer cameras that support RAW image formats often embed the white-balance matrix $T_w$ with the RAW files. With $T_w$, we can obtain the color-correction matrix $T_s$ from $T_c$ ($T_c = T_s \times T_w$). Decomposing the color-correction matrix into two matrices—white-balance and the color space transformation ($T_s$)—can provide several advantages for editing tasks, such as the white balance modification.

According to Kim et al. (2012), the color gamut-mapping *g* can potentially be highly nonlinear and challenging to model using parametric functions. We therefore use scattered point interpolation to model this mapping as done in Kim et al. (2012). These scattered points are sampled from the 3D sRGB color histogram. We examined three different strategies to select the scattered points—namely, uniform sampling, *k*-means clustering, and octree partitioning. The mean values for each partition or cluster are chosen as the control points.

**Fig. 4:**
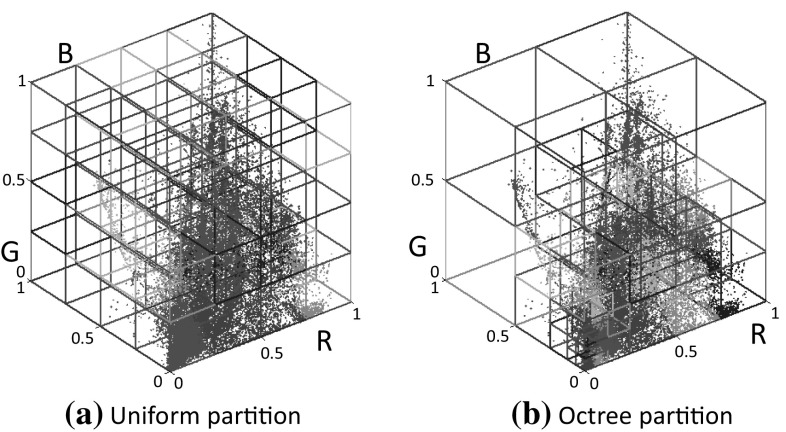
This figure shows an example of partition color space using uniform and octree approaches. The same number of bins $64 = 4^3$ is used for both approaches

It is worth noting that the sRGB and RAW colors in an image are rarely distributed through the whole color space. Therefore, using uniform sampling for the color space has two disadvantages. The first is that many samples are empty and need to be ignored. This makes it hard to know the number of non-empty samples beforehand and therefore challenging to efficiently control the exact number of non-empty samples. Second, the number of colors is not distributed equally, and non-empty samples may not represent a good usage of allocating control points. Figure 4a shows an example using uniform sampling. This also implies that lattice-based methods (e.g., Lin et al. 2012) are also not appropriate for this problem as they attempt to define a lattice over the entire color space. A straightforward solution would be to use *k*-means clustering. The drawback for *k*-means clustering is the required running-time since the number of cluster centers and input colors is relatively large. In this paper, we adapt an octree algorithm proposed by Meagher (1980) to partition the RGB color space. To do so, we introduce a modification to the traditional octree approach that is based on a depth-first search strategy, to instead use a a breadth-first search approach. This breadth-first octree construction allows us to control the number of non-empty partitions and sample more scatter points in dense regions (as shown in Fig. 4b). The details of the octree implementation are presented in the next section.

### Modified Octree Partitioning

The basic approach for octree partitioning is a depth-first search that explores as far as possible along each branch before backtracking. The implementation starts with a single bin surrounding all 3D RGB input points. This bin is recursively subdivided into eight smaller bins. Recursion is stopped when a certain criterion is met. Three common conditions to stop bin subdivision are: (1) a bin contains fewer than a given number of points $\delta _p$; (2) a bin reaches a minimum volume $\delta _l$; (3) a bin reaches a maximum depth $\delta _d$.

However, in our situation, the number of control points is limited to a fixed amount to stay within our 64 KB data limit. Because of this, we need to keep track of the number of non-empty bins and stop the partitioning process when it reaches this fixed number of bins. This is challenging to do with a depth-first search approach because it will first attempt to subdivide the bin to its lowest depth before backtracking to the top-level partitions. As a result, it can quickly allocate all the allowed control points before having a chance to visit other partitions in histogram 3D volume. To overcome this limitation, we modify the octree partitioning scheme to use a breadth-first search strategy. This involves using a queue *Q* to store all the current leaf-nodes. At each iteration, the node at the front of the queue *Q* is extracted and checked for whether it satisfies one of the above stopping conditions. If the nodes need further division, the non-empty sub-bins of the node will be added to the rear of the queue *Q* and the number of non-empty bins is updated. This process will be iterated until it reaches the desired number of bins *K*. By doing this, bins having similar size and similar number of RGB points will be divided a similar number of times. The details for our modified octree partition are given in Algorithm 1. 
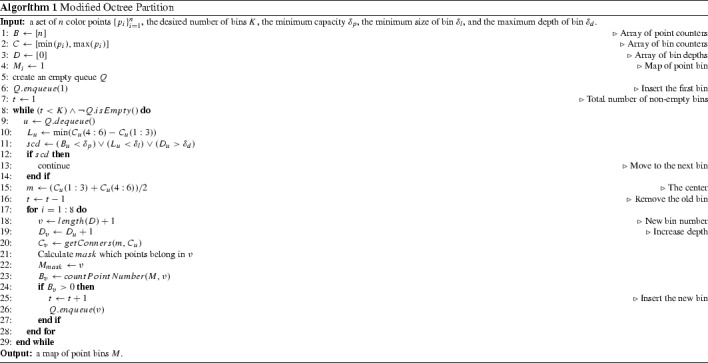


Table 2 shows the comparison among the three strategies to select the scattered points for modeling the gamut-mapping function *g*. Here, the same number of bins ($4096 = 16^3$) is used in all three sampling methods. As can be seen, the running time for uniform sampling is the smallest but its reconstructed errors are highest since the number of non-empty bins is relatively small (around 7%). Using *k*-means can obtain reasonable reconstruction results but the running time is significantly higher. Our modified octree sampling obtains the best reconstructed results and is $10\,\times$ faster than *k*-means clustering. It is worth noting that the octree partitioning may not guarantee the exact number of returned non-empty bins as one or more sub-bin(s) (up to eight) are created at each division. However, it can reach very close to the given number within eight control points.

### Saturation Encoding

The procedure described in Sects. 3.1 and 3.2 targets the modeling of the sRGB and RAW color space relationship. This works directly from the color histograms of the sRGB and RAW image pairs and encodes no spatial information. As a result, this method cannot address situations where RAW values have been clipped or highly compressed when mapping to sRGB. Such clipping commonly happens when the dynamic range of the RAW image exceeds the sRGB’s intensity range and one or more color channels are fully saturated at their maximum value. This is often associated with “overexposure” in certain regions due to bright or highly specular objects in the scene.

**Fig. 5:**
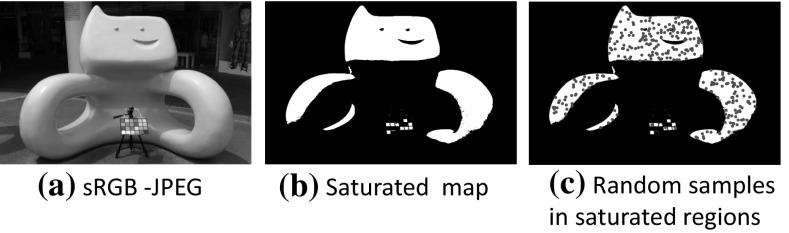
This figure shows an example of saturation encoding. **a** The original sRGB–JPEG image. **b** The saturation map, which is identified from (**a**). **c** Spatial samples locations (red dots) that are randomly sampled in these saturated regions (Color figure online)

To address this issue, we use an approach that stores additional spatial pixel samples for these saturated regions. To this end, a saturated map is first identified by thresholding the sRGB–JPEG image. Any pixel that contains one or more channels larger than the threshold (i.e., $\tau = 252/255$) is considered saturated. We then uniformly sample random spatial locations in these saturated regions to obtain corresponding RAW values (as shown in Fig. 5). This additional information is also kept in the data of the JPEG file as the saturation table, *S*. To avoid having to also save the spatial coordinates of these RAW RGB samples, we ensure that the same random number generator used to gather these samples is used when decoding the data. As a result, this approach allows us to have more samples (nearly double the amount). Note that if an image does not contain large saturated regions, this step can be ignored.

**Table 2:** This table shows the results from three different strategies to select the scattered points for modeling the gamut-mapping function, *g*

	Uniform	K-means	Octree
Begin	4096	4096	4096
Return	283	4096	4091
Time(s)	0.53	101.70	11.18
RMSE	0.0037	0.0026	$\mathbf{0.0023}$

The lowest RMSE is shown in bold

*Note* These are uniform partitioning, k-means clustering, and our octree partitioning. The running time of each of these approaches is given in seconds. The RMSE for the reconstructions obtained with each of these strategies is also shown

### Data Embedding

After the color-mapping parameters are estimated and the saturation table is extracted (if necessary), the parameter values are embedded as data into the JPEG file. The data structure in JPEG contains several segments. Each segment has capacity for a different kind of data. These are delimited by two-byte codes called markers (Brower et al. 2011; Hamilton 1992). One of these segments is the comment (COM) segment. A COM segment does not interfere with the image stored in the JPEG file. The maximum size of a COM segment is 64 KB. However, a JPEG file can support multiple COM segments. In order to fit our data within these COM segments, we have limited our data’s size to be within 128 KB.

**Fig. 6:**
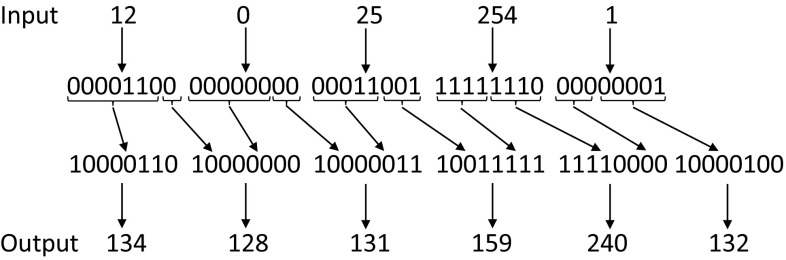
This figure shows an example of our encoding method that avoids null characters

We used one COM segment to store estimated color-mapping data and another COM segment for the saturation table. The JPEG standard *does* allow 0x00 bit sequences to be embedded in the comment field; however, a number of standard libraries do not implement this correctly. Because of this, we have found it a good practice to avoid the special character “null” (i.e., 0x00) as some JPEG decoders interpret this as the end of the text comment.

To avoid the null character in the sequence of characters, we used a simple scheme as follows. The character sequence is converted into a sequence of binary bits. At every seventh bit, an additional bit 1 is inserted. This new bit stream is then converted to ASCII characters. By inserting this additional bit in this periodic manner the COM segment will not contain the null character. Figure 6 shows an example of this approach. By inserting this additional bit, the real storage size of data in the COM segment reduces to 56 KB (7 / 8 of the original size, 64 KB). This is why the data allocation for each part shown in Table 1 is only 56 KB.

### RAW Reconstruction

To reconstruct the RAW values, the data embedded in the JPEG file is first extracted and decoded by converting the text string to a bit string and then removing the additional bit pattern. We now have back all the parameterized data: the inverse white-balance matrix $T_w^{-1}$, the inverse color space transformation $T_c^{-1}$, the control points for the inverse gamut map $g^{-1}$, and the control points for the inverse tone-mapping $f^{-1}$. The RAW values are reconstructed by first applying the inverse tone-mapping $f^{-1}$ to obtain the linearized sRGB image. Then the gamut mapping is applied. We adopt a linear tetrahedral interpolation (Kasson et al. 1993) to model the gamut mapping since the scattered points are in 3D color space. Next, the inverse color space transformation is applied, and finally the white-balance step is undone to obtain the reconstructed RAW image.

For handling saturation, the saturation mask is identified again by thresholding the sRGB–JPEG image. Then the same random number generator is used to reconstruct the spatial coordinates for the RAW values in the saturation table. These ground truth RAW values replace the incorrect values in saturated regions of the reconstructed RAW image.

**Fig. 7:**
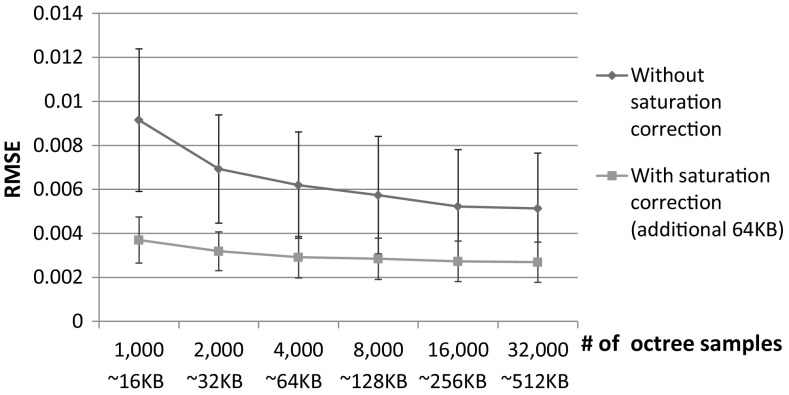
This figure shows the reconstruction errors (in term of RMSE) against different numbers of samples used in the octree sampling. In this experiment, we allow the sampling to go beyond 64 KB and up to 512 KB. The plot shows the results of eight overexposed images where the mean and standard deviation of the RMSE are also reported. Without saturation correction (shown by the blue line), there is a slight improvement when the number of parameters used to model the camera-pixel is increased. However, with only an additional 64 KB of spatial information for saturation correction, the error is significantly improved (shown by the red line). Only small gains are made with the addition of more control points in the octree sampling (Color figure online)

**Fig. 8:**
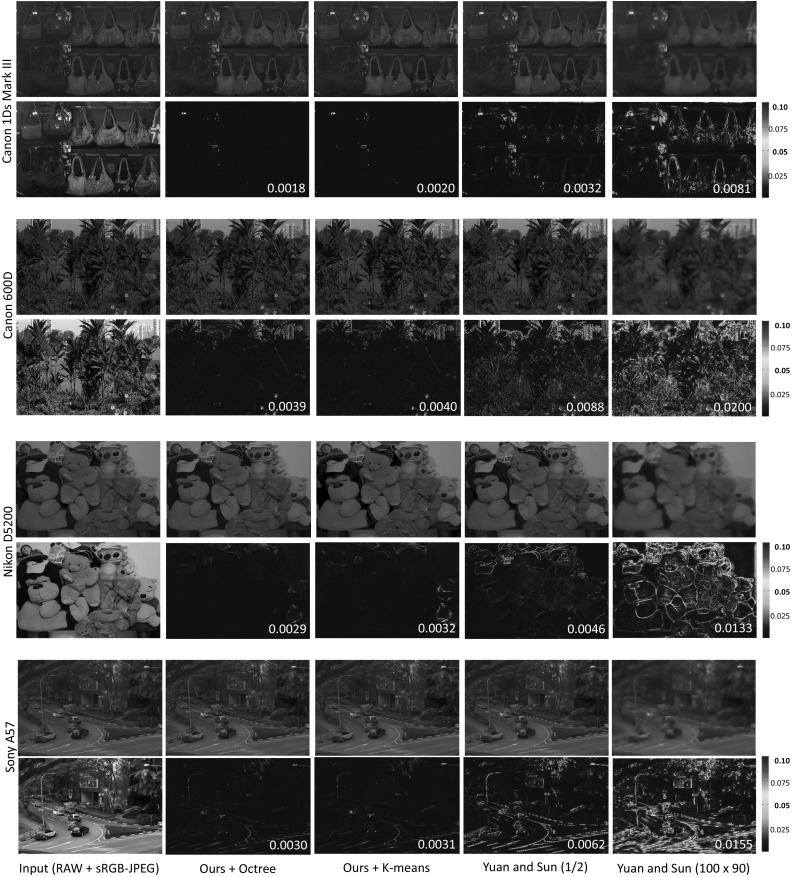
[Without saturation correction] This figure shows results on images that do not require saturation correction and also shows comparisons between our approach and our implementation of the upsampling approach proposed by Yuan and Sun (2011) for various scenes and cameras (a Canon 1Ds Mark III, a Canon 600D, a Nikon D5200, and a Sony $\alpha$57). The RMSEs for each method are shown in the bottom right of each error map

**Table 3:** This table shows the comparison between our method and upsampling method proposed in Yuan and Sun (2011) in terms of RMSE

Camera name	Ours	Yuan and Sun (2011) (1/2)	Yuan and Sun (2011) ($100 \times 90$)
Canon 1Ds	$\varvec{0.0018}$	0.0049	0.0135
Canon 600D	$\varvec{0.0038}$	0.0085	0.0191
Nikon D5200	$\varvec{0.0033}$	0.0078	0.0173
Sony $\alpha$57	$\varvec{0.0020}$	0.0055	0.0150

The lowest RMSE is shown in bold

*Note* For the upsampling method (Yuan and Sun 2011), RAW images at resolutions of half of the original size and $100 \times 90$ are used for upsampling

To correct the other unsampled pixels in the saturated regions, we adopted the upsampling method proposed by Kopf et al. (2007) that uses a joint-bilateral filter strategy (Tomasi and Manduchi 1998). However, in our case these samples are not grid-based; thus we cannot use a normal window as a supporting region for kernels. Instead, we modify the bilaterial upsampling to use the *k*-nearest neighbor pixels in the saturation mask. Specifically, for each pixel in a saturated region, we find the *k*-nearest neighboring pixels stored in the saturation table (in our implementation $k=25$). Using the bilateral filter notation, the range weights are computed using the sRGB values at these *k* sample locations. The spatial weights are computed as the Euclidean distance to these samples’ spatial locations. Using the range and spatial weight, a missing pixel can be computed using the RAW values at these samples as follows:

3 $$\begin{aligned} E_p = \frac{1}{W_p}\sum _{q \in \varOmega }{f(||p - q||) g(||I_p - I_q||) E_q}, \end{aligned}$$

where *f* is the spatial kernel centered over *p* for smoothing differences in coordinates, and *g* is the range filter kernel centered at the image value at *p* for smoothing differences in intensity. The term $\varOmega$ is the spatial support of the kernel *f*, which is *k*-nearest neighboring pixels in our case. The term $W_p$ is a normalizing factor, such that the sum of the weights in *f* and *g* is equal to one.

To appreciate the improvements offered by this saturation step, we compare results obtained with/without saturation correction with different amounts of samples for octree sampling (beyond our restriction of 64 KB). Figure 7 shows the reconstruction errors (in terms of RMSE) against different numbers of samples for octree sampling (the approximate size for storage is shown below). Eight overexposed images are examined and their means and deviations are reported. Without saturation correction (shown by the blue line), there is a slight improvement when the size of the camera-pipeline parameters is increased (even though the total size is up to 512 KB). However, with only an additional 64 KB of spatial information for saturation correction, the error is significantly improved (shown by the red line).

**Fig. 9:**
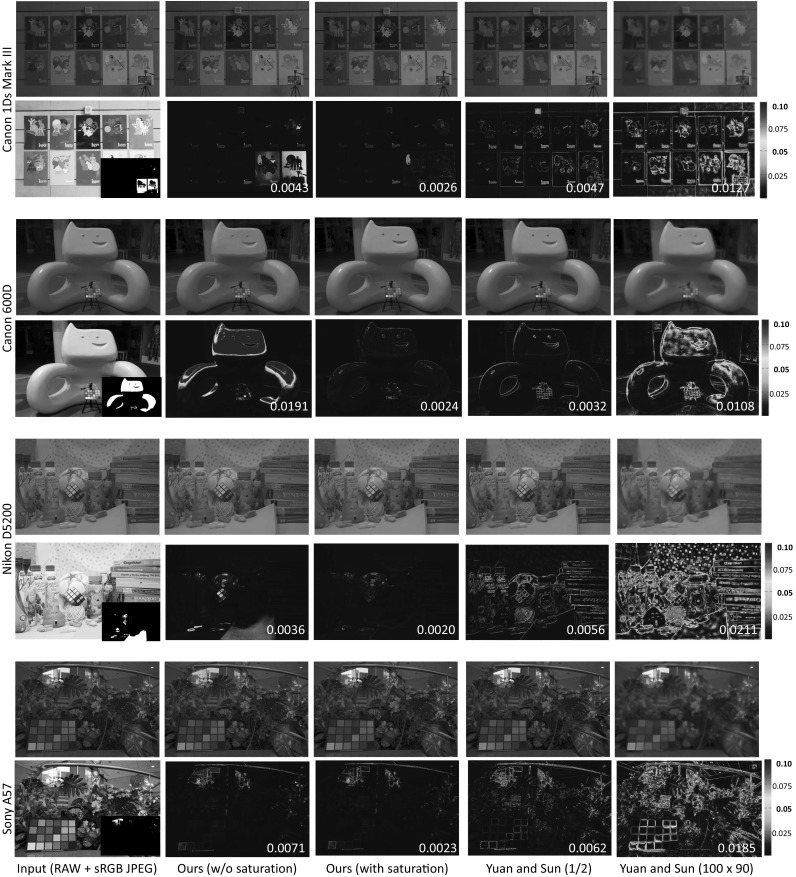
[With saturation correction] This figure shows results on images that do require saturation correction and also shows comparisons between our approach and our implementation of the upsampling approach proposed by Yuan and Sun (2011) for various scenes and cameras (a Canon 1Ds Mark III, a Canon 600D, a Nikon D5200, and a Sony $\alpha$57). The bottom right in the first column is the saturation map where white color indicates saturated pixels. The RMSEs for the each method are shown in the bottom right of each error map

**Fig. 10:**
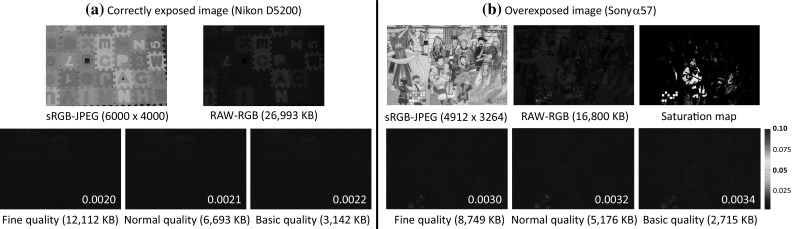
This figure shows two examples of using different qualities of sRGB–JPEG images for reconstructing the RAW-RGB image. **a** Shows correctly exposed image captured by a Nikon D5200. **b** Shows an image with overexposure captured by a Sony $\alpha$57. Here, three different qualities—fine, normal, and basic—are examined. The RMSEs for each quality are shown in the bottom right of each error map

## Results

In this section, we compared our RAW reconstruction strategy with alternative techniques, including our method using *k*-means clustering, our method using the proposed octree method, our method with and without saturation correction, and the upsampling method by Yuan and Sun (2011). Yuan and Sun (2011) do not provide code and we have re-implemented their approach. We note that we have modified their approach to use the recent state-of-the-art method proposed by Ferstl et al. (2013) to perform the image upsampling. Images used in this paper were taken from various online data sets that contain RAW-sRGB pairs, including images from Kim et al. (2012), Cheng et al. (2014), Nguyen et al. (2014), and Dang-Nguyen et al. (2015).

We start with examples that do not require saturation correction. Figure 8 shows the results for images from various cameras and scenes. A jet map is used to show the error between the original and reconstructed RAW image. The root mean square error (RMSE) is also shown to quantitatively evaluate the results. For Yuan and Sun’s method (2011), RAW images at resolutions of half of the original size are used for upsampling. For a fairer comparison, we also used a small RAW image of resolution $100 \times 90$, which can be placed inside 64 KB of data. Table 3 shows the results in terms of RMSE. For each camera, 30 images were examined and the average RMSE was reported. The proposed octree partitioning approach provides RAW reconstructions with the lowest RMSE. Note that the errors shown also include quantization errors in the 8-bit sRGB image, which is around 0.001, or 0.1% in average.

**Fig. 11:**
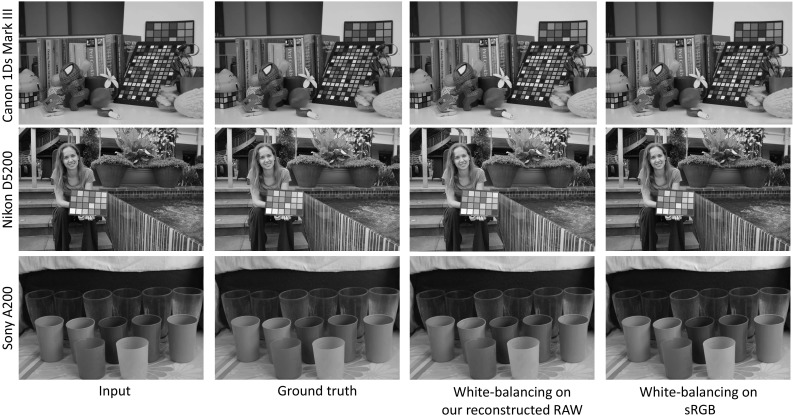
This figure shows examples for correcting white balance for different cameras: a Canon 1Ds Mark III, a Nikon D5200, and a Sony $\alpha$57. The first column is the input images captured under the wrong white-balance settings; the second column shows the ground truth images captured under the proper settings. The third column displays the results obtained by applying white-balance correction using our reconstructed RAW images. The final column shows the results obtained by applying white-balance correction directly on the sRGB–JPEG images

We also test our approach for images where saturation correction is needed. We compared our RAW reconstruction with or without saturation correction, and the upsampling method by Yuan and Sun (2011). Figure 9 shows the results. As can be seen, with an additional 64 KB of RAW values our method can significantly reduce the error in the saturated regions.

**Fig. 12:**
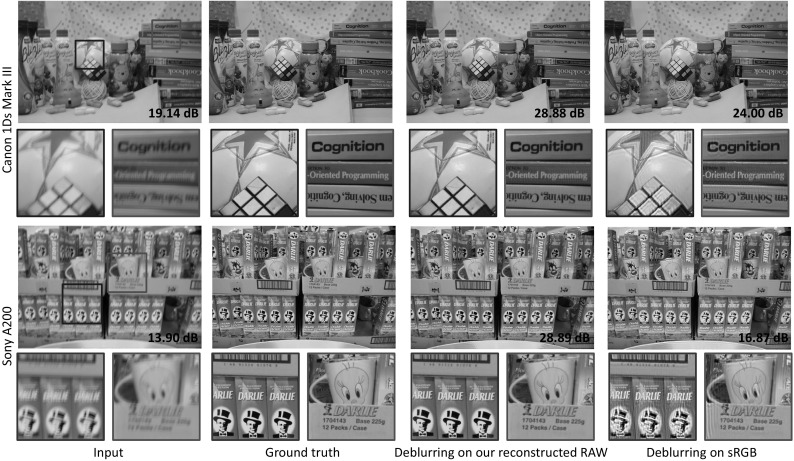
This figure shows examples for image deblurring. A motion blur on the non-blurred ground truth RAW images is performed. The blurred sRGB image is synthesized using the parameterized color pipeline model. We applied our method to reconstruct the blurred RAW image, then deblurred it, and converted it back to the sRGB image. The first and third rows show the results, while the second and fourth rows show close-ups of particular regions. The signal-to-noise ratios (SNRs) are reported at the bottom right of each image

We note that our proposed method does not attempt to remove any compression artifacts that arise due to the lossy nature of JPEG. We assume that the input image is saved as a high-quality JPEG; however, for the sake of completeness, we examine the effect of different JPEG qualities on the reconstructed RAW results. Most digital cameras support three image quality types, fine, normal, and basic, which correspond to the compression ratios (1 / 4, 1 / 8, and 1 / 16). Figure 10 shows two examples: a correctly exposed image captured by a Nikon D5200 and an overexposed image captured by a Sony $\alpha$57. It is clear that the JPEG quality does affect the reconstructed RAW-RGB images; however, the increased error is still within acceptable levels.

**Fig. 13:**
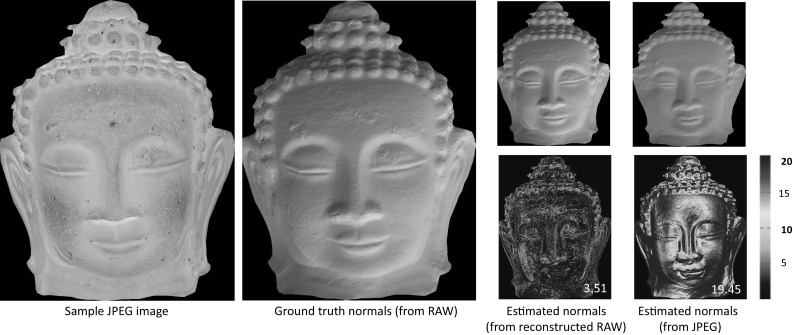
This figure shows an example of our RAW reconstruction for photometric stereo. The first column shows one of the sample sRGB–JPEG images. The second shows the ground truth normals map obtained by applied Quéau et al. (2017) using RAW images. The third and fourth columns display the results (along with the error) obtained from our reconstructed RAW and JPEG images respectively. The angular error maps (in degree) and the mean error are displayed at the bottom

## Applications

We conclude our results section by demonstrating the usefulness of the reconstructed RAW image data with three applications: white balance, image deblurring, and photometric stereo. It is well known that having access to the original RAW image is advantageous for these applications.

### White-Balance Correction

As noted in the processing pipeline in Fig. 2 in Sect. 3, white-balancing is a procedure that is applied early in the processing pipeline. Attempting to change white balance in the sRGB image is challenging as it cannot undo the photo-finishing that has been applied to the sRGB image.

In this experiment, we compared applying white-balance correction on our reconstructed RAW and the original sRGB–JPEG. The comparison results are shown in Fig. 11. The input images are captured under a wrong white-balance setting, while the ground truth images are captured under the proper settings. Here, the achromatic colors on the color checker boards are manually selected to use as the scene illumination color. White-balance correction on the reconstructed RAW images is visually better than correction using the sRGB images.

### Image Deblurring

Image deblurring assumes a linear image formation model in the form $I_B = I \otimes h$, where *I* is the latent image and *h* is a blur kernel. For blurred sRGB images, the relationship is not truly linear between $I_B$ and *I*. Work by Tai et al. (2013) showed that the nonlinear response of the camera changes the shape of the *h* to a spatially varying convolution, making image deblurring even more challenging. Thus, it is desirable to deblur the image in the linear RAW space.

In this application, we compared the deblurring method proposed in Krishnan and Fergus (2009) on our reconstructed RAW and sRGB–JPEG images. This is done by applying a motion blur on a ground truth RAW image and then using the estimated parameters in the camera color pipeline to synthesize the blurred sRGB input images. Figure 12 shows the results of the deblurred sRGB and deblurred reconstructed RAW image. The signal-to-noise ratios (SNRs) are also reported at the bottom right of each image. As can be seen, deblurring of the reconstructed RAW images gives superior results.

### Photometric Stereo

Another vision application that requires access to linear scene radiance data is photometric stereo. This is a computer vision technique for estimating the surface normals of an object by observing the object under different lighting conditions. Specifically, this method assumes that the amount of light reflected by a surface depends on the orientation of the surface in relation to the light source and the observer. Under a Lambertian reflectance assumption, the reflected light intensity can be modelled in the form $I = k(L \cdot n)$, where *I* is the observed intensity; *L* is the light direction; *n* is the surface normal; and *k* is the reflectivity albedo. By measuring the amount of light reflected from different directions of the light sources, the surface normal may be constrained to a single orientation. This application therefore conventionally uses RAW data as input.

In this application, we evaluated applying the photometric stereo method on our reconstructed RAW and sRGB–JPEG images. The RAW images are used to recover surface normals that we treat as ground truth. The code and dataset used are provided from Quéau et al. (2017). Figure 13 shows the angular error maps for the estimated normals using our reconstructed RAW as well as the JPEG images. As can be seen, applying photometric stereo on the reconstructed RAW images produces smaller normal estimate errors than applying on sRGB images.

## Discussion and Conclusion

We have described a method to encode the necessary data with the sRGB image for reconstructing a high-quality RAW image. Our approach produces a fully self-contained, 100% compliant JPEG file that can be used as-is, not affecting any existing image workflows. This method can reconstruct the original RAW to within 0.5% error with only 64 KB of overhead for camera-pipeline parameters and an additional 64 KB for saturation correction. Although we focused only on the JPEG compression format as it is the most popular format for storing sRGB images, we believe that the proposed encoding technique can be easily extended to other formats that can support the inclusion of metadata (e.g., PNG).

One drawback of our method is that we have optimized our framework to minimize error for backward mapping from sRGB to RAW; however, for many photography tasks (e.g., our white-balance example), the forward mapping from RAW back to sRGB is needed. A future topic would be to modify our method to consider the bidirectional reconstruction error. Also, while our method provides better results in the saturated region than prior work, there are still notable errors in these regions, especially about boundaries. Improving the results in these areas is a topic suitable for future work.
